# The impact of job stress on perceived professional benefits among Chinese nurses caring for patients with gynecological cancer: mediating effects of perceived social support and self-efficacy

**DOI:** 10.3389/fpsyg.2024.1344185

**Published:** 2024-04-03

**Authors:** Yuxin Zhang, Xinhai Meng, Lihua Zhou

**Affiliations:** School of Nursing, Anhui Medical University, Hefei, China

**Keywords:** gynecological cancer, job stress, nurses, perceived social support, perceived professional benefits, self-efficacy

## Abstract

**Introduction:**

Nurses caring for patients with gynecological cancer experience significant job stress, which adversely impacts their mental health. Previous studies have indicated that perceived professional benefits serves as a protective factor for nurses’ mental health, and factors such as job stress, perceived social support and self-efficacy influence their perceived professional benefits. However, the relationships between these factors and the associated mechanisms have remained incompletely understood. This study explored the role of perceived social support and self-efficacy in job stress and perceived professional benefits among nurses caring for patients with gynecological cancer.

**Methods:**

During June and July 2023, an investigation was conducted in Anhui Province. The Nurse Job Stressors Scale, Perceived Social Support Scale, Nurses’ Perceived Professional Benefits Questionnaire and General Self-Efficacy Scale were administered to 311 nurses caring for patients with gynecological cancer. A chained-mediated effect model was constructed and validated.

**Results:**

Job stress negatively affected nurses’ perceived professional benefits. Perceived social support was a mediator in job stress and nurses’ perceived professional benefits, with a mediating effect value of −0.093. Additionally, perceived social support and self-efficacy functioned as sequential mediators in this relationship, with a mediating effect value of −0.032.

**Conclusion:**

This study unveils the influencing mechanisms of job stress on perceived professional benefits of nurses caring for patients with gynecological cancer. It is essential for nursing managers to alleviate nurses’ job stress, provide sufficient and effective social support and improve their self-efficacy, ultimately enhancing their perceived professional benefits.

## 1 Introduction

Gynecological cancers, malignancies of the female reproductive system, impose a heavy health burden on women worldwide (Jiang et al., 2018; Doddi et al., 2023). Based on the 2020 Global Cancer Statistics Report, there were approximately 1.39 million new incidences of gynecological cancers worldwide (Sung et al., 2021). In China, in 2020, there were approximately 250,000 new cases of gynecological cancers, and the number kept rising (Cao et al., 2021). The survival rates for patients with gynecological cancer (GC) have improved with the use of early screening and optimized treatments, resulting in more survivors of gynecological cancers. However, this also indicates a rising number of cases of gynecological cancers in China (Feng et al., 2021).

Nurses are recognized as major providers of professional support and expertise for patients with GC, addressing their multifaceted care needs during the diagnosis, treatment, survivorship, or end-of-life stages (Beesley et al., 2018, 2019; Skorstad et al., 2022). Prior study has indicated that nurses caring for patients with GC face job-related challenges and stressful events. For example, Cook et al. (2021) found that their roles are evolving and expanding. Sun et al. (2023) observed that nurses commonly encounter challenges in delivering sufficient supportive care to patients with GC and Buckley et al. (2018) indicated that they need to keep learning to meet the evolving care requirements of patients with GC. In addition, previous studies have shown that patients with GC often experience emotional distress, including anxiety, depression, and mania, with prevalence rates ranging from 40.6 to 66% (Aquil et al., 2021; Suskin et al., 2022; Lee et al., 2023). Nurses caring for them are frequently exposed to these negative emotions over the long term, which can lead to psychological stress and adversely affect their mental health (Edlund et al., 2023).

Furthermore, previous studies have pointed out that GC being a female-specific type of cancer, it brings about a series of disease-specific and treatment-related side effects, such as advancing menopause, decreased quality of sexual life, deterioration of the couple’s relationship, and loss of fertility (Beesley et al., 2018). This means that patients with GC not only have general supportive care needs similar to those of patients with cancer in general, but also have additional specific supportive care needs (Beesley et al., 2018), such as the need for special symptom management, the need to strengthen their couple relationship, and to maintain dignity (Sun et al., 2023). Given the increasing incidence of GC, coupled with the high job demands it places on nurses due to its nature as a female-specific disease, the potentially high levels of job stress nurses face may result in professional mental health issues like compassion fatigue, burnout and emotional exhaustion (Lupo et al., 2013; Al-Ruzzieh and Ayaad, 2021; Edlund et al., 2023). Considering the essential supportive role of nurses for patients with GC in quality of life, symptom management, and care satisfaction (Cook et al., 2015; Beesley et al., 2019), it is important to effectively alleviate their professional mental health problems. With the rise of positive psychology in the nursing field, researchers have turned their attention to the facilitation of positive professional emotional experiences (Hu and Liu, 2013).

Nurses’ perceived professional benefits (NPPB) refers to the benefits that nurses perceive their nursing work brings to them (Hu and Liu, 2013). It is a positive emotional experience about the nursing profession and serves as an internal personal motivational factor that contributes to nurses’ professional development (Wang et al., 2023). Previous study has shown that NPPB is beneficial for mental health (Zhang et al., 2022). Specifically, NPPB can mitigate several adverse outcomes of the nursing environment, such as anxiety, depression, and job burnout (Zhang et al., 2022; Zou and Xie, 2022). Zhan et al.'s (2020) study found that NPPB can contribute not just to nurses’ professional development, but to their subjective well-being too. Relevant studies have indicated that NPPB can enhance nurses’ job satisfaction, improve their willingness to stay in the job, and reduce the turnover rate, which is essential to alleviating the nursing shortage (Zhao et al., 2017). Considering the aforementioned findings, it is crucial to explore the mechanisms by which influence NPPB among nurses caring for patients with GC.

Job stress arises from the interplay between the work environment and individuals’ roles within their jobs (Richardson and Rothstein, 2008). Nurses often face high job-related stress (Luan et al., 2017), such as moral distress, complex and difficult decision-making, high workload, and facing patient death (Eche et al., 2023; Jeong et al., 2023). Previous research has observed a notable negative relationship between job stress and NPPB (Chen et al., 2020). High-load and high-stress work environments may lead to negative emotions among nurses, and reduce work motivation and satisfaction, thereby decreasing NPPB (Chen et al., 2020). However, a systematic review of factors influencing NPPB found that not all nurses experience such a decrease when faced with job stress. Some experience growth, which promotes an increase in NPPB (Sun and Liang, 2021). Nevertheless, the inner mechanisms for this are unclear, and it has not been verified whether other variables influence the connection between job stress and NPPB among nurses caring for patients with GC. Thus, we put forward hypothesis 1:

Hypothesis 1: Job stress is significantly associated with NPPB.

Perceived social support is an important psychological resource that pertains to the emotional experience that individuals feel when they perceive support, respect, and understanding in society (Sarason et al., 1991; Zhang et al., 2024). It encompasses how individuals perceive and interpret different social relationships, indicating the practical use of social support (Geuzinge et al., 2020). Previous research suggests a negative association between job stress and perceived social support (Su, 2019). When nurses are affected by job stress, perceived social support serves as a vital and protective psychological resource that enhances their adaptability to stressful situations, and facilitates prompt and effective recovery from stressful experiences (Su, 2019). Therefore, we propose hypothesis 2:

Hypothesis 2: Job stress is negatively related to perceived social support.

Previous research has also indicated that perceived social support is positively associated with NPPB (Zhang et al., 2021). Nurses with high perceived social support are more attuned to the availability of social support they receive, and are more inclined to perceive support from diverse sources including family, friends, and colleagues, which is essential to enhance their professional identity, professional worth, and job satisfaction (Zhao et al., 2021). Furthermore, the main effect model of social support implies that social support positively impacts nurses’ mental well-being and sustains positive emotional experiences (Cohen and Wills, 1985). Thus, nurses who perceive higher levels of social support are more likely to access and effectively utilize this resource within their network, which is beneficial for improving their NPPB. Therefore, we put forward hypothesis 3, 4:

Hypothesis 3: Perceived social support is positively associated with NPPB.

Hypothesis 4: The connection between job stress and NPPB is mediated by perceived social support.

The Cognitive Appraisal Theory of Stress helps explore the association between job stress and stress reactions. It emphasizes that an individual’s cognitive appraisal of stressful events directly influences their psychological responses (Lazarus and Folkman, 1984). Self-efficacy is one commonly observed cognitive appraisal that may mediate the relationship between job stress and psychological reactions (Dianat et al., 2021). It involves individuals’ evaluations of their ability to perform particular tasks and their confidence in successfully executing specific behaviors (Bandura, 1977). It is dynamic and can evolve through learning, gaining experience, and receiving feedback (Ammentorp et al., 2007). Previous research demonstrated that job stress has a negative influence on nurses’ self-efficacy (Cabrera-Aguilar et al., 2023). This suggests that as nurses feel less control over situations and have lower self-efficacy, job stress tends to rise. Self-efficacy may influence how individuals cognitively, emotionally, and strategically respond to the stress experienced in their job (Tsang et al., 2012). Nurses with high self-efficacy can cope with job stress more effectively due to their belief in their own abilities (Cabrera-Aguilar et al., 2023). They can maintain emotional stability and experience positive emotions (Lu et al., 2023), often viewing job stress as a challenge rather than a threat. Consequently, they are less susceptible to the deleterious influence of job stress (Lu et al., 2023). Therefore, hypothesis 5 is proposed:

Hypothesis 5: Job stress is negatively related to self-efficacy.

Meanwhile, a previous study found that self-efficacy is positively correlated with NPPB (Li et al., 2021). Empirical study shows that self-efficacy is closely associated with positive states (Xanthopoulou et al., 2007). Nurses with high self-efficacy possess positive outcome expectations and exhibit greater work engagement. They feel more energized and focused, take pride in their work, and find it meaningful. This leads to higher levels of personal and professional satisfaction, ultimately improving NPPB (De Simone et al., 2018; Pérez-Fuentes et al., 2018; Li et al., 2021). In summary, according to the cognitive appraisal theory of stress mentioned above, we hypothesize that self-efficacy may mediate the relationship between job stress and psychological reactions (NPPB). Accordingly, we propose hypothesis 6, 7:

Hypothesis 6: Self-efficacy is positively related to NPPB.

Hypothesis 7: Self-efficacy acts as a mediator between job stress and NPPB.

Previous research has shown that perceived social support is positively correlated with self-efficacy (Hu et al., 2018; Lu et al., 2023). This implies that perceived support from significant others can serve as a resource for developing and fostering one’s efficacy beliefs regarding performance, functioning, and coping with challenges (Liu and Aungsuroch, 2019). In addition, self-efficacy can be enhanced by external empathy and help. Social support through emotional outreach, information exchange, and interpersonal relationships provide resources and also help to enhance self-efficacy (Bandura, 1986). Research indicates that perceived social support and self-efficacy are useful psychological coping resources that work together to promote mental health and well-being (Liu and Aungsuroch, 2019; Lu et al., 2023). For instance, Wang et al.'s (2018) study indicates that perceived social support can enhance nurses’ self-efficacy, subsequently boosting their resilience. Lu et al. (2023) discovered that perceived social support positively affects the psychological well-being of nurses with low self-efficacy. High perceived social support provides nurses with psychological resources to cope with job stress, strengthen their belief in overcoming challenges, and recognize the value and meaning of their work, thereby increasing job satisfaction and NPPB. Therefore, drawing from the prior studies, we put forward hypothesis 8, 9:

Hypothesis 8: Perceived social support is positively related to self-efficacy.

Hypothesis 9: Perceived social support and self-efficacy are sequential mediators linking job stress and NPPB.

**Figure 1:**
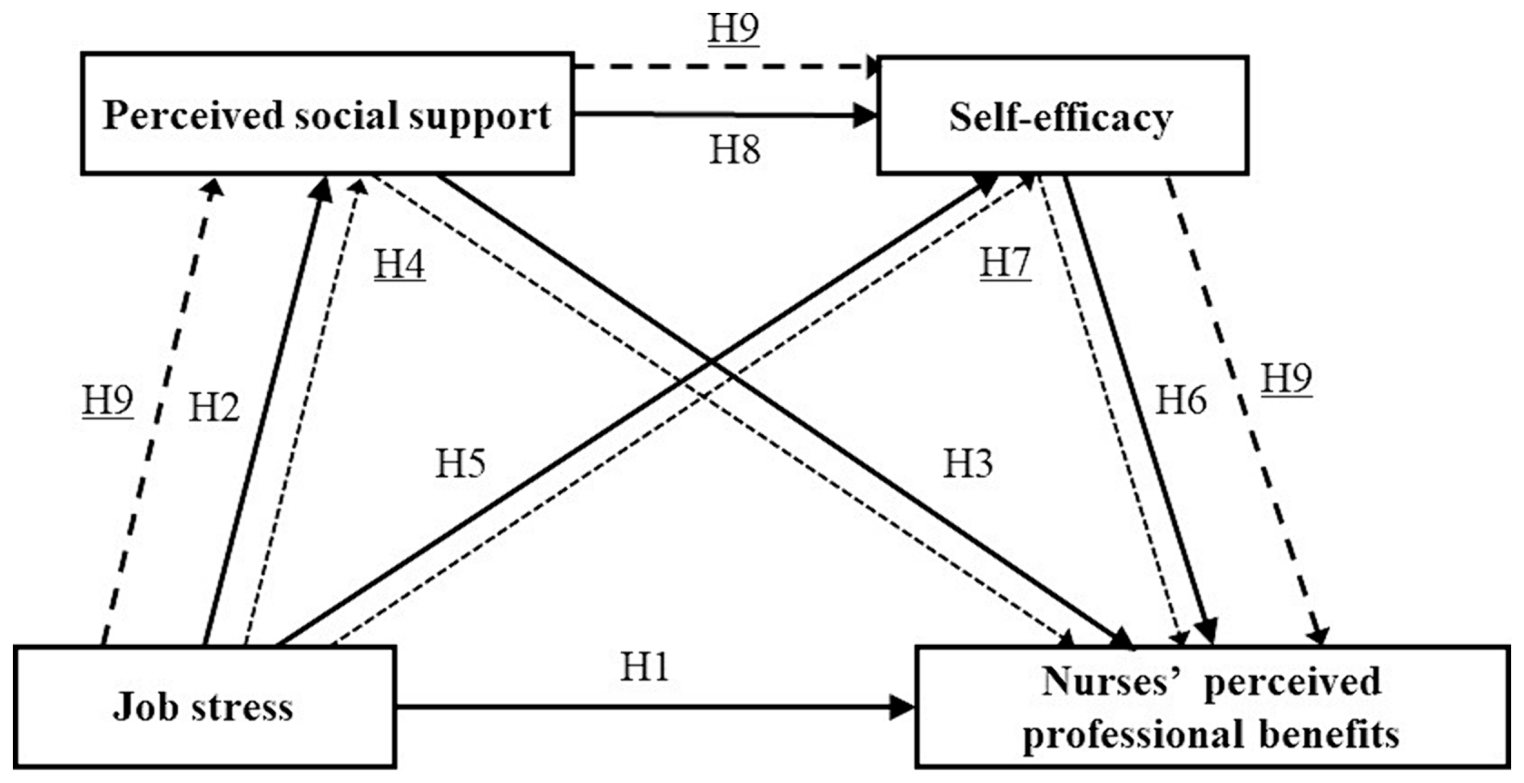
Hypothetical model diagram.

In summary, NPPB as a positive psychological resource is critical for protecting nurses’ mental health and deserves further exploration. While previous research has established a link between job stress and it (Chen et al., 2020; Sun and Liang, 2021), the specific mechanisms that drive this connection are still not fully understood. It has remained unclear whether perceived social support and self-efficacy play a mediating role in the relationship between these two factors. To bridge this knowledge gap, our study constructed a chain mediation model diagram to elucidate the influencing mechanisms between the four variables, aiming to provide nursing managers with evidence to develop personalized strategies that improve NPPB and foster nurses’ mental health. (see Figure 1).

## 2 Materials and methods

### 2.1 Study design, participants

In this cross-sectional study, based on the major geographical subregions of the Anhui Province, we used convenience sampling and conducted the investigation in the gynecology wards of a total of 22 tertiary hospitals located in 10 cities in the following subregions: South (Huangshan, Tongling, Wuhu), Central (Anqing, Chuzhou, Hefei, Lu’an), and North (Bozhou, Fuyang, Suzhou). A total of 311 nurses caring for patients with GC were enlisted. Inclusion criteria were: (a) being a registered nurse providing care during the diagnosis, treatment, survivorship, or end-of-life stages of patients with GC; (b) informed consent to participate; (c) having worked for at least 1 year. We excluded (a) nurse interns, advanced practice nurses and (b) nurses off duty during the investigation. The G*Power software was used to calculate the sample size, the significance level α = 0.05, the statistical power 1- β was set to 0.95. The calculated sample size of 119, factoring in a 20% attrition rate, necessitated a minimum of 149 participants. This study included a collective of 311 participants, satisfying the required sample size criteria.

### 2.2 Data collection

We contacted the head nurses of gynecology wards in the 22 tertiary hospitals, providing them with relevant information about the study and obtained their consent. We used Wenjunxing, a commonly utilized online investigation platform in China, for investigation and data collection. We sent the head nurses of the gynecology wards with a QR code for accessing the electronic questionnaire via WeChat (an instant messaging communication tool) and requested them to forward it to the potential participants. The QR code could be scanned by the participants with their mobile phones to enter the questionnaire filling interface. We set the first page of the questionnaire as an informed consent form, participants were guaranteed anonymity in the study, and informed of their right to withdraw at any point. Furthermore, they were promised that the collected data would be kept confidential. After reading the informed consent form and selecting the “Agree” option at the bottom of the page, participants could begin answering the questionnaires. All questions were set as mandatory, and the questionnaires could only be submitted successfully if all questions were answered; the same IP address could only respond once. A total of 318 questionnaires were collected, with 311 deemed valid (effective response rate 97.8%).

### 2.3 Instruments

#### 2.3.1 Socio-demographic data

This section included age, gender, education level, marital status, number of children, working position, work experience in caring for patients with GC, professional title and forms of employment.

#### 2.3.2 Nurse job stressors scale

This scale was developed by Li and Liu (2000) and was used to evaluate nurses’ levels of job stress. It comprises a total of 35 items across five dimensions: nursing profession and job, workload and time allocation, work environment and resources, management and interpersonal relations, and patient care. A four-point Likert scale (“1-completely disagree” to “4-completely agree”) was used. Within the scale, scores can vary from 35 to 140, with higher scores reflecting higher job stress. In this study, the Cronbach’s α coefficient was 0.961.

#### 2.3.3 Nurses’ perceived professional benefits questionnaire

A 33-item instrument was compiled by Hu and Liu (2013) and comprises five dimensions: self-growth, good nurse–patient relationship, recognition from family, relatives and friends, positive occupational perception, and sense of belonging to a team. Each item was scored by five-point scale (“1-completely disagree” to “5-completely agree”). Scores on the scale range from 33 to 165, with higher scores indicating higher NPPB. The Cronbach’s α coefficient for this study was 0.981.

#### 2.3.4 Perceived social support scale

This scale was compiled by Zimet et al. (1988) and the translation of the Chinese version was conducted by Jiang (2001). This scale was adapted to evaluate nurses’ levels of perceived social support. The instrument comprises 12 items, distributed across three dimensions: family support, friends support, and others support. Each item was scored by seven-point scale (“1-very strongly disagree” to “7-very strongly agree”). The total score obtained ranged from 12 to 84. The higher the score, the more social support nurses perceived. The Cronbach’s α coefficient for this study was 0.957.

#### 2.3.5 Self-efficacy scale

The Chinese version of this scale was conducted by Zhang and Schwarzer (1995). This instrument was used to evaluate nurses’ confidence in their capacity to handle difficult situations, obstacles, and setbacks. The scale is a single-dimensional measure comprising of 10 items, which are scored by four-point scale (“1-completely incorrect” to “4-completely correct”). The overall score falls within the range of 10 to 40, with higher scores associated with stronger self-efficacy. The Cronbach’s α coefficient for this study was 0.947.

### 2.4 Data analysis

SPSS 26.0 was used for statistical data analysis, including descriptive statistics analysis, independent samples *t*-tests or one-way analysis of variance, and Pearson’s correlation analysis was conducted between both factors of NPPB, perceived social support, job stress, and self-efficacy. And the PROCESS macro (Model 6) was adapted to test the chain mediation model. The *p* < 0.05 indicates statistical significance.

## 3 Results

### 3.1 Socio-demographic characteristics

The present investigation involved 311 nurses, with ages ranging from 23 to 58 years. The vast majority of them were women (98.4%), 90.4% of participants had a bachelor’s degree or higher, 78.1% were married, and more than half had at least one child (74%). Specific information about the participants can be found in Table 1.

**Table 1:** Socio-demographic characteristics and differences in the perceived professional benefits scores of nurses.

Variables	Frequency(percentage)	Mean(range)	Perceived professional benefits(M ± SD)	*F/t*	*p*
Age(years)		34.28 (23–58)		8.889	<0.001
21 ~ 30	102 (32.8)		127.95 ± 18.86		
31 ~ 40	154 (49.5)		132.31 ± 17.95		
41 ~ 50	46 (14.8)		142.78 ± 20.32		
51 ~ 60	9 (2.9)		148.33 ± 15.49		
Gender				−1.418	0.157
Male	5 (1.6)		120.80 ± 26.16		
Female	306 (98.4)		133.09 ± 19.11		
Education level				3.024	0.050
Junior college	30 (9.6)		132.26 ± 19.15		
Undergraduate	277 (89.1)		132.62 ± 19.15		
Graduate or above	4 (1.3)		156.25 ± 16.83		
Marital status				3.119	0.002
Unmarried/others	68 (21.9)		126.54 ± 19.56		
Married	243 (78.1)		134.67 ± 18.83		
Number of children				6.922	0.001
0	81 (26.0)		126.24 ± 18.71		
1	138 (44.4)		135.78 ± 17.43		
≥2	92 (29.6)		134.40 ± 21.05		
Work experience (years)		10.07 (1–38)		6.459	<0.001
1 ~ 10	187 (60.1)		129.56 ± 17.80		
11 ~ 20	94 (30.2)		135.90 ± 19.53		
21 ~ 30	25 (8.1)		143.72 ± 22.75		
31 ~ 40	5 (1.6)		146.80 ± 17.92		
Working position				16.850	<0.001
Nursing manager	32 (10.3)		149.50 ± 15.85		
Teaching nurse	63 (20.3)		135.28 ± 15.98		
General nurse	216 (69.4)		129.73 ± 19.28		
Professional title				8.607	<0.001
Primary nurse	122 (39.2)		128.83 ± 18.27		
Intermediate nurse	175 (56.3)		134.42 ± 19.20		
Senior nurse	14 (4.5)		149.07 ± 18.15		
Forms of employment				3.398	0.035
Contract employed nurse	189 (60.8)		132.38 ± 18.09		
Personal agent nurse	75 (24.1)		130.20 ± 19.11		
Formal employed nurse	47 (15.1)		139.23 ± 19.25		

### 3.2 Control variables

Table 1 also presents the score differences of NPPB across different socio-demographic characteristics. Based on this information, we considered factors such as age, marital status, working position, work experience in caring for patients with GC, number of children, professional title and forms of employment as control variables.

### 3.3 Descriptive and correlation analysis of investigation variables

The results presented that job stress was negatively correlated with perceived social support, self-efficacy and NPPB; perceived social support, self-efficacy and NPPB were positively correlated with each other in pairs (see Table 2).

**Table 2:** The statistical descriptions and associations among the main variables.

Variables	M ± SD	1	2	3	4
1. Nurses’ perceived professional benefits	4.02 ± 0.58	–			
2. Perceived social support	5.24 ± 0.97	0.593**	–		
3. Self-efficacy	2.66 ± 0.61	0.521**	0.436**	–	
4. Job stress	2.56 ± 0.47	−0.344**	−0.269**	−0.125*	–

**p < 0.01, *p < 0.05.

### 3.4 Chain mediation analysis

**Figure 2:**
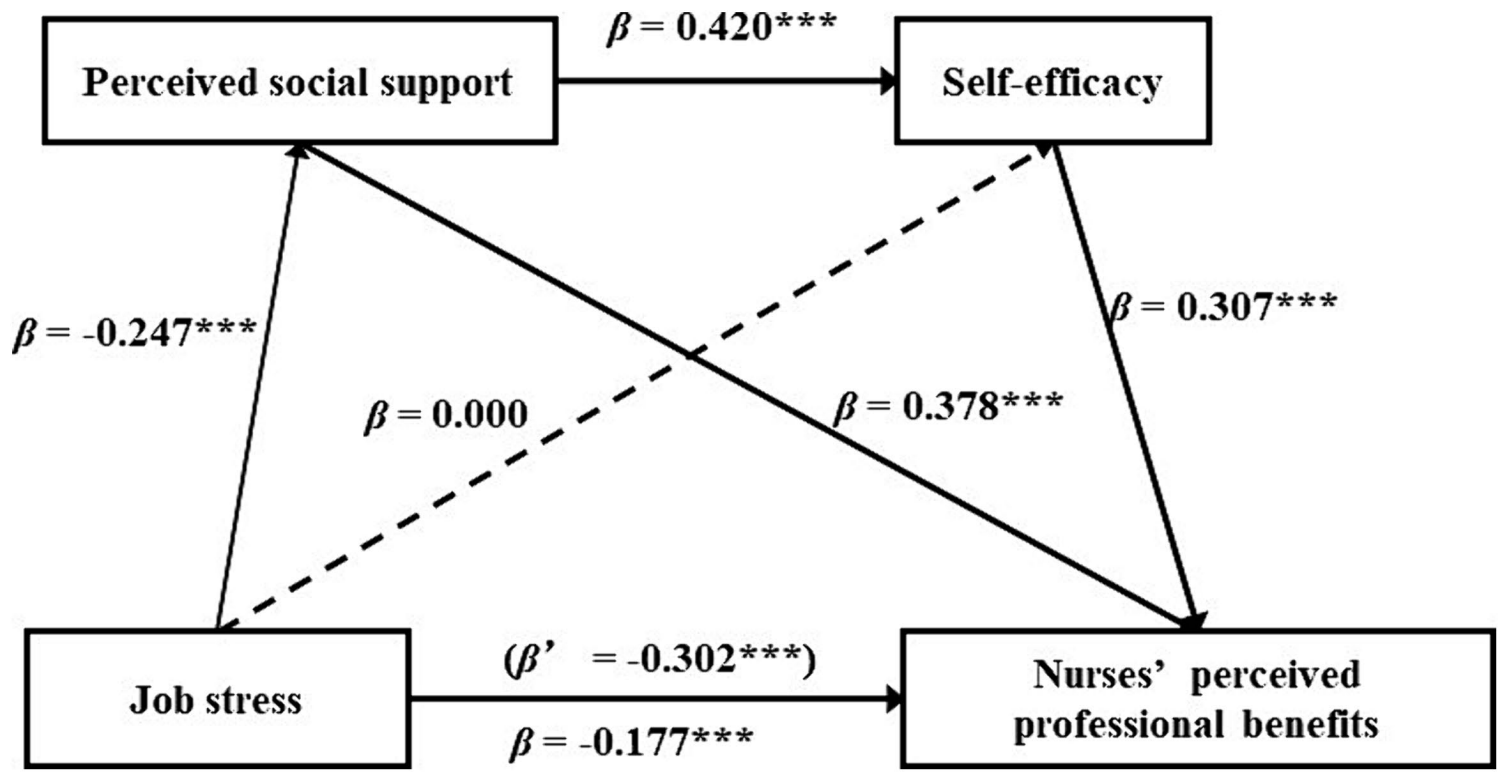
Chain mediation model diagram of job stress on nurses’ perceived professional benefits. ***p < 0.001.

Model 6 in the PROCESS was employed to test the chain mediation between job stress and NPPB, while controlling for control variables. The results showed that job stress negatively impacted NPPB (*β’ =* −0.302, *p <* 0.001), the impact remained significant (*β =* −0.177, *p <* 0.001) after including perceived social support and self-efficacy in the regression equation. Job stress negatively influenced perceived social support (*β =* −0.247, *p <* 0.001); while having no effect on self-efficacy (*β =* 0.000, *p >* 0.05). Perceived social support positively influenced self-efficacy (*β =* 0.420, *p <* 0.001); and both perceived social support and self-efficacy positively influenced NPPB (respectively, *β =* 0.378, *p <* 0.001; *β =* 0.307, *p <* 0.001). Therefore, hypothesis 1, 2, 3, 6, 8 were supported, while hypothesis 5 was not. (see Table 3 and Figure 2).

**Table 3:** Regression analysis among variables.

Variables	*β*	*t*	*p*	LLCI	ULCI	*R^2^*	*F*
Result variable: perceived social support							
Predictor variable: job stress	−0.247	−4.383	<0.001	−0.358	−0.136	0.098	4.102
Result variable: self-efficacy							
Predictor variable: job stress	0.000	−0.009	>0.05	−0.107	0.106	0.217	9.263
Mediator perceived social support	0.420	7.812	<0.001	0.314	0.525		
Result variable: nurses’ perceived professional benefits							
Predictor variable: job stress	−0.177	−4.178	<0.001	−0.260	−0.093	0.525	33.184
Mediator 1 perceived social support	0.378	8.219	<0.001	0.287	0.468		
Mediator 2 self-efficacy	0.307	6.831	<0.001	0.219	0.396		
Result variable: nurses’ perceived professional benefits							
Independent variable job stress	−0.302	−5.762	<0.001	−0.405	−0.199	0.220	10.645

The mediating effect was examined by means of the bootstrap method (after 5,000 repeated samples, 95% confidence intervals were estimated) (Table 4). The results showed that the direct impact of job stress on NPPB constituted 58.6% of the total effect, whereas the indirect influence comprised 41.4% of the total effect, the indirect effect was 30.8% for path 1 and 10.6% for path 3, indicating that job stress indirectly affected NPPB through two significant pathways, the indirect path1(indirect effect = −0.093) (job stress → perceived social support →NPPB); the indirect path 3 (indirect effect = −0.032) (job stress → perceived social support →self-efficacy→ NPPB). However, the indirect path 2 (job stress →self-efficacy→ NPPB), where 95% CI contained 0, this indirect path was not significant. Thus, hypothesis 4 and 9 were supported, whereas hypothesis 7 was not.

**Table 4:** Bootstrap analysis of the chain mediating model.

Path	Effect	Boot SE	Boot	Boot	Effect ratio
			LLCI	ULCI	
Total effect	−0.302	0.052	−0.405	−0.199	100%
Direct effect	−0.177	0.042	−0.260	−0.093	58.6%
Total indirect effect	−0.125	0.041	−0.204	−0.044	41.4%
Path1	−0.093	0.025	−0.145	−0.045	30.8%
Path2	0.000	0.021	−0.041	0.041	0.0%
Path3	−0.032	0.010	−0.052	−0.014	10.6%
Comparsion1 (Path1 and Path2)	−0.093	0.029	−0.150	−0.034	
Comparsion2 (Path1 and Path3)	−0.061	0.022	−0.109	−0.023	
Comparsion3 (Path2 and Path3)	0.032	0.022	−0.011	0.076	

Path1: Job stress → Perceived social support → Nurses’ perceived professional benefits; Path2: Job stress → Self-efficacy → Nurses’ perceived professional benefits; Path 3: Job stress → Perceived social support → Self-efficacy → Nurses’ perceived professional benefits.

## 4 Discussion

The current study elucidated the roles of perceived social support and self-efficacy in the association between job stress and NPPB. Job stress not only directly and negatively influences NPPB, but also indirectly influences it through the mediating role of perceived social support and the chain mediation of perceived social support and self-efficacy.

This research reveals that job stress directly and negatively influences NPPB, confirming Hypothesis 1, in accordance with previous research findings (Chen et al., 2020). Job stress arises when an individual’s capacity to manage work tasks does not align with the demands of their job (Li and Liu, 2000). When caring for patients with GC, nurses play multiple roles simultaneously, including advisor, guide, key contact, and team support provider, and must switch between these roles at any given time to meet the evolving needs of patients (Kobleder et al., 2017). Under such circumstances, the job demands may exceed the nurse’s capacity, resulting in a decrease in focus, a diminished recognition of the advantages and motivation for work, and an adverse emotional encounter within professional practice (Yang et al., 2020). In addition, nurses also experience psychological stress, including exposure to the negative emotions of patients and their partners (Camara et al., 2019), and caring for patients with terminal gynecological cancer (Boz and Teskereci, 2021). As a result, they must not only balance a demanding work environment, but also manage the negative emotions that may arise in their work (Kase et al., 2019). This dual challenge may lead to adverse mental health outcomes for nurses who have prolonged contact with patients with GC, making them more susceptible to compassion fatigue leading to negative professional experiences such as insomnia and depression (Edlund et al., 2023). This requires nursing managers to appropriately increase the staffing levels of nurses in gynecology wards, to share the multiple roles of caring for patients with GC; to conduct regular stress assessments of nurses’ job stressors and develop measures to reduce these; in addition, they should pay more attention to nurses’ mental health, provide psychological counseling services, and conduct structured training programs to reduce their psychological distress, increasing their NPPB.

Firstly, perceived social support positively impacts NPPB, and acts as an intermediary in the connection between job stress and NPPB; this mediator accounted for 30.8% of the total effect, supporting Hypothesis 2, 3, 4. Similar to previous studies (Liu and Aungsuroch, 2019; Su, 2019; Zhang et al., 2021;). These findings may be plausibly explained by the buffering model of social support, which suggests that when faced with job stress, social support serves as a buffer, alleviating its adverse impacts, promoting mental health, and sustaining positive experiences (Cohen and Wills, 1985). Thus, when nurses caring for patients with GC encounter stressful events such as excessive workload, work–family conflict, and exposure to negative emotions, support from family, colleagues, leaders, and significant others can provide resources and assistance (Su, 2019). Nurses with high perceived social support tend to utilize these resources more effectively and are inclined to adopt positive strategies to cope with job stress, thereby inhibiting or buffering its adverse effects, and helping nurses sustain positive emotional experiences (Cohen and Wills, 1985; Sarason et al., 1991; Xiang et al., 2017). Additionally, their work of caring for patients with GC is respected, praised, and affirmed by their families, friends, leaders, colleagues, and significant others, contributing to positive career perceptions, which are essential for enhancing NPPB (Hu and Liu, 2014; Polat and Terzi, 2021; Sen and Yildirim, 2023). Therefore, nursing managers should provide systematic organizational support to help nurses cope with the job stress faced while caring for patients with GC, establish flexible scheduling to allow them to build social support networks with friends and family amidst their busy work, encourage them to participate in GC-related charitable activities to gain support from others, and encourage nurses to share their work experiences with relatives and family members to benefit from their understanding and support. Managers should also implement measures like mindfulness interventions to improve nurses’ perception and understanding of social support, which can help them identify positive aspects of their work and alleviate the psychological distress and heavy workload.

Secondly, the mediation of self-efficacy between job stress and NPPB was found to be insignificant, contrary to the study of Cabrera-Aguilar et al. (2023). Hypothesis 5 and 7 were therefore not supported. Successful experiences are crucial for self-efficacy development (Bandura, 1986). The nurses we analyzed in this study had around 10 years of experience caring for GC patients. They had accumulated a wealth of work experience and accomplishments, having established a high and consistent sense of self-efficacy, they could effectively manage job stress, so the usual tasks of caring for patients with GC did not significantly affect their self-efficacy. However, the present study only examined the level of job stress and did not consider the severity and duration of the stressful events; ultimately, the mediating role of self-efficacy between job stress and NPPB may not be fully ascertainable.

Thirdly, this study offers novel insights into how perceived social support and self-efficacy mediate the association between job stress and NPPB, confirming hypothesis 6, 8, 9. Similar to prior research (Li et al., 2021; Lu et al., 2023). Nurses with high perceived social support are more inclined to perceive and accept social support when experiencing job stress while caring for patients with GC. And they are better at using social support to manage job stress (Cohen and Wills, 1985; Geuzinge et al., 2020). Meanwhile, nurses maintain contact with their family, friends, colleagues, and leaders, perceiving support in various social relationships (Sarason et al., 1991). This not only contributes to positive emotional experiences and mastery of alternative experiences, but also stimulates social persuasion, all of which contribute to improving self-efficacy (Bandura et al., 1997). Bandura et al. (1997) maintained that self-efficacy is crucial for motivation, behavior, and personal development. Individuals with high self-efficacy believe that positive outcomes can be achieved through problem solving and goal setting, and they often use problem-focused coping strategies (Cabrera-Aguilar et al., 2023). The study of Zhou et al. (2023) suggested the effectiveness of such strategies for nurses facing challenges in caring for patients with GC. To address challenges, nurses have employed various coping strategies, such as implementing evidence-based practices through critical utilization of new knowledge and enhancing their ability to communicate effectively (Kobleder et al., 2017). In addressing challenges and delivering care, nurses find fulfillment and value in supporting patients with GC, which in turn improves their NPPB (Zhou et al., 2023). Therefore, nursing managers should provide their staff with positive feedback and support, encourage nurses experienced in caring for patients with GC to assist their younger peers, and offer learning opportunities to enhance their nursing capability and confidence, ultimately enhancing their NPPB.

## 5 Conclusion

This study found that job stress, perceived social support, and self-efficacy directly or indirectly influence NPPB. Job stress negatively affects NPPB. Perceived social support can mediate the relationship between job stress and NPPB independently, while the combined mediation of perceived social support and self-efficacy also plays a role in how job stress affects NPPB. This paper presents new findings and offers a fresh perspective on enhancing the NPPB among nurses caring for patients with GC. It is essential for nursing managers to alleviate nurses’ job stress, provide sufficient and effective social support and improve their self-efficacy, ultimately enhancing their NPPB.

### 5.1 Limitations

Our study had certain limitations. Firstly, its cross-sectional design constrains our capacity to establish causal relationships; further research can validate these results through longitudinal research. Secondly, this study utilized online self-reporting questionnaires for data collection; as such, recall and selection biases may have occurred and may have led to errors in accuracy and reliability. Thirdly, since participants volunteered to take part, there was a non-response bias. In addition, it was not possible to determine the reasons for refusal to participate in the investigation. In the future, various investigation methods should be offered to enhance participants engagement. Finally, this study only investigated hospitals within the Anhui Province, limiting the sample’s representativeness and the generalizability of the study findings. Future research could involve conducting multi-sample investigations across multiple regions.

## Data availability statement

The raw data supporting the conclusions of this article will be made available by the authors, without undue reservation.

## Ethics statement

The studies involving humans were approved by the current study received approval from the Ethics Committee of Anhui Medical University (ethical approval number: 84230015). The studies were conducted in accordance with the local legislation and institutional requirements. Written informed consent for participation was not required from the participants or the participants’ legal guardians/next of kin in accordance with the national legislation and institutional requirements.

## Author contributions

YZ: Writing – review & editing, Writing – original draft, Visualization, Validation, Supervision, Software, Resources, Project administration, Methodology, Investigation, Funding acquisition, Formal analysis, Data curation, Conceptualization. XM: Writing – original draft, Software, Data curation. LZ: Writing – review & editing, Validation, Supervision, Methodology.
