# Supplementary material for: The impact of job stress on perceived professional benefits among Chinese nurses caring for patients with gynecological cancer: mediating effects of perceived social support and self-efficacy
Source: Front Psychol. 2024 Apr 3;15:1344185. doi: 10.3389/fpsyg.2024.1344185 (PMC11021783; doi:10.3389/fpsyg.2024.1344185)
Supplement: Supplementary file 2 [file Data_Sheet_2.docx]

护理妇科癌症患者护士职业获益感研究

一、一般资料

1. 年龄（请填写数字，如28）
2. 性别：🞎男 🞎女
3. 学历：🞎大专 🞎本科 🞎研究生及以上
4. 婚姻状态：🞎已婚 🞎未婚 🞎离异 🞎丧偶
5. 孩子数目：🞎0 🞎1 🞎2 🞎3个及以上
6. 从事与妇科癌症患者相关护理工作的工龄：请填写数字，如6）
7. 职务：🞎护士长及以上 🞎带教老师 🞎护士
8. 职称：🞎护士 🞎护师 🞎主管护师 🞎副主任护师及以上
9. 人事关系: 🞎合同制 🞎人事代理 🞎在编

二、请认真阅读下面的每个条目，并决定该陈述对您适用的程度，请您根据自己的实际情况，选择最符合的选项。

| 1.护理工作的社会地位太低 | 1-非常不同意 | 2-不同意 | 3-同意 | 4-非常同意 |
| --- | --- | --- | --- | --- |
| 2.继续深造的机会太少 | 1-非常不同意 | 2-不同意 | 3-同意 | 4-非常同意 |
| 3.工资及其他的福利待遇低 | 1-非常不同意 | 2-不同意 | 3-同意 | 4-非常同意 |
| 4.晋升的机会太少 | 1-非常不同意 | 2-不同意 | 3-同意 | 4-非常同意 |
| 5.经常倒班 | 1-非常不同意 | 2-不同意 | 3-同意 | 4-非常同意 |
| 6.工作中的独立性少 | 1-非常不同意 | 2-不同意 | 3-同意 | 4-非常同意 |
| 7.工作分工不明确 | 1-非常不同意 | 2-不同意 | 3-同意 | 4-非常同意 |
| 8.护士的工作量太大 | 1-非常不同意 | 2-不同意 | 3-同意 | 4-非常同意 |
| 9.上班的护士数量少 | 1-非常不同意 | 2-不同意 | 3-同意 | 4-非常同意 |
| 10.没有时间对病人实施心理护理 | 1-非常不同意 | 2-不同意 | 3-同意 | 4-非常同意 |
| 11.非护理性的工作太多 | 1-非常不同意 | 2-不同意 | 3-同意 | 4-非常同意 |
| 12.无用的书面工作太多 | 1-非常不同意 | 2-不同意 | 3-同意 | 4-非常同意 |
| 13.工作环境差 | 1-非常不同意 | 2-不同意 | 3-同意 | 4-非常同意 |
| 14.工作中所需的仪器设备不足 | 1-非常不同意 | 2-不同意 | 3-同意 | 4-非常同意 |
| 15.病区拥挤 | 1-非常不同意 | 2-不同意 | 3-同意 | 4-非常同意 |
| 16.担心工作中出现差错事故 | 1-非常不同意 | 2-不同意 | 3-同意 | 4-非常同意 |
| 17.护士工作未被病人及家属承认 | 1-非常不同意 | 2-不同意 | 3-同意 | 4-非常同意 |
| 18.护理的病人病情过重 | 1-非常不同意 | 2-不同意 | 3-同意 | 4-非常同意 |
| 19.病人的家属不礼貌 | 1-非常不同意 | 2-不同意 | 3-同意 | 4-非常同意 |
| 20.病人的要求太高或太过分 | 1-非常不同意 | 2-不同意 | 3-同意 | 4-非常同意 |
| 21.病人不礼貌 | 1-非常不同意 | 2-不同意 | 3-同意 | 4-非常同意 |
| 22.病人不合作 | 1-非常不同意 | 2-不同意 | 3-同意 | 4-非常同意 |
| 23.所学知识不能满足病人及家属心理需求 | 1-非常不同意 | 2-不同意 | 3-同意 | 4-非常同意 |
| 24.缺乏病人教育的有关知识 | 1-非常不同意 | 2-不同意 | 3-同意 | 4-非常同意 |
| 25.担心护理操作会引起病人的疼痛 | 1-非常不同意 | 2-不同意 | 3-同意 | 4-非常同意 |
| 26.护理的病人突然死亡 | 1-非常不同意 | 2-不同意 | 3-同意 | 4-非常同意 |
| 27.缺乏其他卫生工作人员的理解及尊重 | 1-非常不同意 | 2-不同意 | 3-同意 | 4-非常同意 |
| 28.护理管理者的理解与支持不够 | 1-非常不同意 | 2-不同意 | 3-同意 | 4-非常同意 |
| 29.护理管理者的批评过多 | 1-非常不同意 | 2-不同意 | 3-同意 | 4-非常同意 |
| 30.医生对护理工作过分挑剔 | 1-非常不同意 | 2-不同意 | 3-同意 | 4-非常同意 |
| 31.同事之间缺乏理解与支持 | 1-非常不同意 | 2-不同意 | 3-同意 | 4-非常同意 |
| 32.与护理管理者发生冲突 | 1-非常不同意 | 2-不同意 | 3-同意 | 4-非常同意 |
| 33.与病区的某些护士很难共事 | 1-非常不同意 | 2-不同意 | 3-同意 | 4-非常同意 |
| 34.与医生发生冲突 | 1-非常不同意 | 2-不同意 | 3-同意 | 4-非常同意 |
| 35.同事之间缺乏友好合作的气氛 | 1-非常不同意 | 2-不同意 | 3-同意 | 4-非常同意 |

三、请您根据自己的实际情况，选择最符合的选项。

| 1.在我出现问题时有些人（领导、亲戚、同事）会出现在我的身旁 | 1-极不同意 | 2-很不同意 | 3-稍不同意 | 4-中立 | 5-稍同意 | 6-很同意 | 7-极同意 |
| --- | --- | --- | --- | --- | --- | --- | --- |
| 2.我能与有些人（领导、亲戚、同事）共享快乐与忧愁 | 1-极不同意 | 2-很不同意 | 3-稍不同意 | 4-中立 | 5-稍同意 | 6-很同意 | 7-极同意 |
| 3.我的家庭能够切实具体地给我帮助 | 1-极不同意 | 2-很不同意 | 3-稍不同意 | 4-中立 | 5-稍同意 | 6-很同意 | 7-极同意 |
| 4.在需要时我能够从家庭获得感情上的帮助和支持 | 1-极不同意 | 2-很不同意 | 3-稍不同意 | 4-中立 | 5-稍同意 | 6-很同意 | 7-极同意 |
| 5.当我有困难时有些人（领导、亲戚、同事）是安慰我的真正源泉 | 1-极不同意 | 2-很不同意 | 3-稍不同意 | 4-中立 | 5-稍同意 | 6-很同意 | 7-极同意 |
| 6.我的朋友们能真正的帮助我 | 1-极不同意 | 2-很不同意 | 3-稍不同意 | 4-中立 | 5-稍同意 | 6-很同意 | 7-极同意 |
| 7.在发生困难时我可以依靠我的朋友们 | 1-极不同意 | 2-很不同意 | 3-稍不同意 | 4-中立 | 5-稍同意 | 6-很同意 | 7-极同意 |
| 8.我能与自己的家庭谈论我的难题 | 1-极不同意 | 2-很不同意 | 3-稍不同意 | 4-中立 | 5-稍同意 | 6-很同意 | 7-极同意 |
| 9.我的朋友们能与我分享快乐与忧愁 | 1-极不同意 | 2-很不同意 | 3-稍不同意 | 4-中立 | 5-稍同意 | 6-很同意 | 7-极同意 |
| 10.在我的生活中有些人（领导、亲戚、同事）关心着我的感受 | 1-极不同意 | 2-很不同意 | 3-稍不同意 | 4-中立 | 5-稍同意 | 6-很同意 | 7-极同意 |
| 11.我的家庭能心甘情愿协助我做出各种决定 | 1-极不同意 | 2-很不同意 | 3-稍不同意 | 4-中立 | 5-稍同意 | 6-很同意 | 7-极同意 |
| 12.我能与自己的朋友们谈论我的难题 | 1-极不同意 | 2-很不同意 | 3-稍不同意 | 4-中立 | 5-稍同意 | 6-很同意 | 7-极同意 |

1. 请您仔细阅读每一个句子，根据您内心真实的看法和感受，选择最符合的选项。

| 1.如果我尽力去做的话，我总是能够解决问题的 | 1-完全不正确 | 2-有点正确 | 3-多数正确 | 4-完全正确 |
| --- | --- | --- | --- | --- |
| 2.即使别人反对我，我仍有办法取得我所要的 | 1-完全不正确 | 2-有点正确 | 3-多数正确 | 4-完全正确 |
| 3.我自信能有效地应付任何突如其来的事情 | 1-完全不正确 | 2-有点正确 | 3-多数正确 | 4-完全正确 |
| 4.对我来说，坚持理想和达成目标是轻而易举的 | 1-完全不正确 | 2-有点正确 | 3-多数正确 | 4-完全正确 |
| 5.以我的才智，我定能应付意料之外的情况 | 1-完全不正确 | 2-有点正确 | 3-多数正确 | 4-完全正确 |
| 6.如果我付出必要的努力，我一定能解决大多数的难题 | 1-完全不正确 | 2-有点正确 | 3-多数正确 | 4-完全正确 |
| 7.我能冷静地面对困难，因为我信赖自己处理问题的能力 | 1-完全不正确 | 2-有点正确 | 3-多数正确 | 4-完全正确 |
| 8.面对一个难题时，我通常能找到几个解决方法 | 1-完全不正确 | 2-有点正确 | 3-多数正确 | 4-完全正确 |
| 9.有麻烦的时候，我通常能想到些应付的方法 | 1-完全不正确 | 2-有点正确 | 3-多数正确 | 4-完全正确 |
| 10.无论什么事在我身上发生，我都能够应对自如 | 1-完全不正确 | 2-有点正确 | 3-多数正确 | 4-完全正确 |

五、您从护士职业中感受到哪些益处和收获？请根据您的实际情况选择相应的选项，答案没有对错之分。

| 1.护士职业的就业机会多且工作稳定 | 1-很不同意 | 2-较不同意 | 3-不确定 | 4-较同意 | 5-很同意 |
| --- | --- | --- | --- | --- | --- |
| 2.家人理解我的工作的特点和社会价值，更加关心、照顾我 | 1-很不同意 | 2-较不同意 | 3-不确定 | 4-较同意 | 5-很同意 |
| 3.在工作中我能得到同事或下属的信任和支持 | 1-很不同意 | 2-较不同意 | 3-不确定 | 4-较同意 | 5-很同意 |
| 4.患者对我的工作表示理解和满意，是对我的一种鼓励 | 1-很不同意 | 2-较不同意 | 3-不确定 | 4-较同意 | 5-很同意 |
| 5.带教工作中师生间教学相长促使我进步 | 1-很不同意 | 2-较不同意 | 3-不确定 | 4-较同意 | 5-很同意 |
| 6.从事护士职业使我心胸宽广，更懂得包容他人 | 1-很不同意 | 2-较不同意 | 3-不确定 | 4-较同意 | 5-很同意 |
| 7.护士职业给人“白衣天使”、“救死扶伤”的美好形象，使我感到自豪 | 1-很不同意 | 2-较不同意 | 3-不确定 | 4-较同意 | 5-很同意 |
| 8.亲友因获益于我的职业而非常支持我的工作 | 1-很不同意 | 2-较不同意 | 3-不确定 | 4-较同意 | 5-很同意 |
| 9.护士职业使我获得了自我照护和预防保健的能力 | 1-很不同意 | 2-较不同意 | 3-不确定 | 4-较同意 | 5-很同意 |
| 10.能在工作中为患者提供帮助，我感到很开心 | 1-很不同意 | 2-较不同意 | 3-不确定 | 4-较同意 | 5-很同意 |
| 11.专业能力获得他人的认可，是我成长的动力 | 1-很不同意 | 2-较不同意 | 3-不确定 | 4-较同意 | 5-很同意 |
| 12.家人生病时，我可为他们提供专业指导（包括就医、用药等） | 1-很不同意 | 2-较不同意 | 3-不确定 | 4-较同意 | 5-很同意 |
| 13.成功抢救患者生命后得到患者及其家人的赞许或感激使我提升了职业价值感 | 1-很不同意 | 2-较不同意 | 3-不确定 | 4-较同意 | 5-很同意 |
| 14.职业使我能为亲友提供便利的医疗资源 | 1-很不同意 | 2-较不同意 | 3-不确定 | 4-较同意 | 5-很同意 |
| 15.患者能理解我的工作，令我欣慰 | 1-很不同意 | 2-较不同意 | 3-不确定 | 4-较同意 | 5-很同意 |
| 16.与同事之间的相互交流可增强我的信心和力量 | 1-很不同意 | 2-较不同意 | 3-不确定 | 4-较同意 | 5-很同意 |
| 17.看到患者饱受病痛折磨，我更珍惜生命、知足所有 | 1-很不同意 | 2-较不同意 | 3-不确定 | 4-较同意 | 5-很同意 |
| 18.随着专业能力不断提高，我的职业心态日渐成熟 | 1-很不同意 | 2-较不同意 | 3-不确定 | 4-较同意 | 5-很同意 |
| 19.护士职业给我带来了稳定、有保障的收入 | 1-很不同意 | 2-较不同意 | 3-不确定 | 4-较同意 | 5-很同意 |
| 20.我所在工作团队能互相帮助、相处融洽，使我感到温暖 | 1-很不同意 | 2-较不同意 | 3-不确定 | 4-较同意 | 5-很同意 |
| 21.护士职业使我获得了医学知识、医疗资源 | 1-很不同意 | 2-较不同意 | 3-不确定 | 4-较同意 | 5-很同意 |
| 22.在工作中我能得到领导的指导和肯定 | 1-很不同意 | 2-较不同意 | 3-不确定 | 4-较同意 | 5-很同意 |
| 23.家人生病时，我可为他们提供专业照护 | 1-很不同意 | 2-较不同意 | 3-不确定 | 4-较同意 | 5-很同意 |
| 24.护士职业使我更加认真严谨、有责任感 | 1-很不同意 | 2-较不同意 | 3-不确定 | 4-较同意 | 5-很同意 |
| 25.护士职业能让我发挥所长，实现自身的社会价值 | 1-很不同意 | 2-较不同意 | 3-不确定 | 4-较同意 | 5-很同意 |
| 26.患者在我的护理下好转／康复，给我带来成就感 | 1-很不同意 | 2-较不同意 | 3-不确定 | 4-较同意 | 5-很同意 |
| 27.工作组织可给我提供较多的专业学习机会，让我觉得受到重视和培养 | 1-很不同意 | 2-较不同意 | 3-不确定 | 4-较同意 | 5-很同意 |
| 28.护士职业促使我与各种人打交道，增强了我的人际能力 | 1-很不同意 | 2-较不同意 | 3-不确定 | 4-较同意 | 5-很同意 |
| 29.工作中常遇到突发状况，提升了我的心理素质水平（如协调、应变能力等） | 1-很不同意 | 2-较不同意 | 3-不确定 | 4-较同意 | 5-很同意 |
| 30.我给家人传播了健康保健知识，帮助他们养成良好的生活习惯 | 1-很不同意 | 2-较不同意 | 3-不确定 | 4-较同意 | 5-很同意 |
| 31.我觉得做一名护士挺好的 | 1-很不同意 | 2-较不同意 | 3-不确定 | 4-较同意 | 5-很同意 |
| 32.能在工作中与患者良好沟通、和睦相处，令我感到愉悦 | 1-很不同意 | 2-较不同意 | 3-不确定 | 4-较同意 | 5-很同意 |
| 33.护士职业使我养成了耐心细致的作风 | 1-很不同意 | 2-较不同意 | 3-不确定 | 4-较同意 | 5-很同意 |
